# First-Time Mothers’ and Fathers’ Developmental Changes in the Perception of Their Daughters’ and Sons’ Temperament: Its Association With Parents’ Mental Health

**DOI:** 10.3389/fpsyg.2020.02066

**Published:** 2020-08-20

**Authors:** Cristina Sechi, Laura Vismara, Luca Rollè, Laura Elvira Prino, Loredana Lucarelli

**Affiliations:** ^1^Department of Pedagogy, Psychology, Philosophy, University of Cagliari, Cagliari, Italy; ^2^Department of Psychology, University of Turin, Turin, Italy

**Keywords:** postnatal parental anxiety, postnatal parental depression, infant temperament, gender differences, longitudinal study

## Abstract

**Objective:**

Most studies investigating the role of parenting behaviors on a child’s development are directed to mothers. However, recent analyses show that mothers and fathers have a different influence on a child’s functioning, specifically her/his temperament. The present study explored the developmental change of parents’ perception of their daughters’ and sons’ temperament and its association with parental mental health problems.

**Methods:**

The sample included 188 parents (94 couples) and their at-term 94 babies (55.3% boys, 44.7% girls). Assessments by self-reports were conducted at 3 (Time 1) and 12 (Time 2) months after the children’s birth; at Time 1, mothers and fathers independently answered: the State–Trait Anxiety Inventory (STAI), the Edinburgh Postnatal Depression Scale (EPDS), and the Infant Behavior Questionnaire (IBQ-R). At Time 2, EPDS, STAI, and IBQ-R were again administered to mothers and fathers.

**Results:**

In general, mothers and fathers would give similar descriptions of their child’s temperament throughout the first year of life; however, infant temperament showed developmental changes as well as gender differences. Mother and father anxiety and depression symptoms are associated with the infants’ negative affectivity. Also, mothers with high anxiety and depression levels perceive their infants with a minor tendency to approach novelty, to seek environmental stimulation, and to express/experience positive emotions.

**Conclusion:**

The results highlight the need to screen for infants’ temperament vulnerabilities in the context of maternal and paternal depression in order to protect the child from behavioral, cognitive, and emotional difficulties and to create specific programs aimed at preventing dysfunctional parent–infant relationships.

## Introduction

Temperament may be defined “as the infant’s threshold for positive and negative reactivity and the intensity of its reaction to stimuli” (Austin et al., 2005, p.184), and it is assumed to be biologically based, although it is also shaped by environmental experiences (Zentner and Bates, 2008; Montirosso et al., 2011).

Difficult temperament is characterized by negative affect, feeding and sleeping problems, sensory difficulties, and trouble in adjusting to novelty (Henderson and Wachs, 2007; Zentner and Bates, 2008). These features seem to constitute a risk for later emotional and behavioral problems (Bates, 2001; Guerin et al., 2003; Posner and Rothbart, 2007).

It has been demonstrated that perinatal maternal anxiety and depression are related to infants’ higher stress sensitivity and inadequate affective and cognitive regulation (Essex et al., 2002; Austin et al., 2005; Coplan et al., 2005; Pesonen et al., 2004; Hammarberg et al., 2008).

Furthermore, it has been proven that depressed mothers tend to perceive their babies as difficult, possibly due to their trouble in understanding their infants’ signals (Schuetze and Zeskind, 2001; McGrath et al., 2008).

Most studies have targeted maternal perinatal anxiety and depression; however, a growing body of research indicates that fathers’ anxiety and depression is also relevant for the child’s development (Edhborg et al., 2005; van den Berg et al., 2009; Hohmann-Marriott, 2011). In fact, fathers with self-reported depression, like mothers, would also perceive their children as fussier and more difficult (Atella et al., 2003; Davé et al., 2005). Potapova et al. (2014) confirmed these findings, highlighting the relevance of paternal internalizing problems, parenting-related stress, and infant temperament for their child’s emotional and behavioral regulation capacity.

However, several scholars showed differences in the rates of child temperament on behalf of mothers and fathers. Huynh et al. (2014), for instance, showed that only maternal depression was directly linked with more difficult temperament, whereas paternal depression was significantly related with their child’s difficult temperament only in the presence of maternal depression.

Differences also emerge with respect to the infants’ gender. Else-Quest et al. (2006) meta-analysis indicated that – in children from 3 months to 13 years of age – *inhibitory control* and *perceptual sensitivity* are significantly higher among girls, whereas *activity* and *high-intensity pleasure* are higher among boys; no gender difference emerged in *negative affectivity*.

With respect to fathers, Crick and Zahn–Waxler (2003) demonstrated that fathers’ *surgency* predicted *surgency* at 4 months only for girls, whereas fathers’ *parenting competence* predicted *regulation/orienting* at 6 months only for boys. In addition, fathers’ reports seem to be more influenced by their child’s gender than mothers’ (Parade and Leerkes, 2008; Bayly and Gartstein, 2013). Indeed, Snow et al. (1983) found that fathers support and engage more with their daughters. Instead, fathers are mostly involved in developing early regulation in their infant boys (Schoppe-Sullivan et al., 2006).

Accordingly to the above the literature, the aims of this study were to:

(a) Evaluate differences between fathers’ and mothers’ postnatal anxiety and depression symptoms and the perception of infant temperament.
(b) Evaluate, separately for mothers and fathers, the developmental change in infant temperament and if the perception of child temperament is different with respect to the infants’ gender.
(c) Examine whether mother’s and father’s postnatal anxiety and depression, assessed at 3 and 12 months postpartum (Time 1 and Time 2), are associated with perceived infant’s temperament (Time 1 and Time 2).

### Participants

The study participants were 188 parents (94 couples) and their 94 healthy babies (55.3% boys, 44.7% girls). Of the parents, 78.7% were married couples, and 21.3% were cohabiting; 6.4% of the mothers and 17% of the fathers had an elementary school qualification, 66% of the mothers and 63.8% of the fathers had a high-school qualification, and 27.7% of the mothers and 19.1% of the fathers a college degree. Mothers’ mean age ranged from 26 to 42 years (*M*_*Age*_ = 34.9 years, *SD* = 3.6 years), and fathers’ mean age ranged from 27 to 55 years (*M*_*Age*_ = 38.3 years, *SD* = 5 years). The median income of the parents belonged to the Italian middle working class and socioeconomic status as assessed by a detailed questionnaire and according to the Istituto Nazionale di Statistica classification (ISTAT, 2013). No participant was undergoing medical/psychological treatment at the time of assessment.

### Measures

#### Parental Depression

To measure depression symptoms, we utilized the **Edinburgh Postnatal Depression Scale** (EPDS; Cox et al.1987). It is a self-report form containing 10 items focused on depression symptoms happening within the previous 7 days. The overall score is computed by adding items, each scored on a four-point Likert scale. The adopted cutoff score was >8/9, as recommended by the Italian validated translation (Benvenuti et al., 1999).

In the current study, the internal consistency coefficient for the mothers was α = 0.80 at Time 1 and α = 0.82 at Time 2; for the fathers, it was α = 0.77 at Time 1 and α = 0.75 at Time 2.

#### Parental Anxiety

To measure anxiety symptoms, we used the **State–Trait Anxiety Inventory** (STAI; Spielberger et al., 1983). It is a commonly used self-report form for trait and state anxiety. STAI has 20 items for evaluating trait anxiety (STAI-T) and 20 for state anxiety (STAI-S). The items are evaluated on a four-point Likert scale. The adopted cutoff score was >40, as recommended by the Italian validated translation (Pedrabissi and Santinello, 1989).

In the current study, the internal consistency coefficient for STAI-S was α = 0.89 at Time 1 and α = 0.90 at Time 2 for the mothers; for the fathers, it was α = 0.92 at Time 1 and α = 0.88 at Time 2. The internal consistency coefficient for STAI-T was α = 0.88 at Time 1 and α = 0.84 at Time 2 for the mothers; for the fathers, it was α = 0.91 at Time 1 and α = 0.87 at Time 2.

#### Child Temperament

To measure child temperament, we used the **Infant Behavior Questionnaire** [IBQ-R; Gartstein and Rothbart, 2003; Italian Validation by Montirosso et al. (2011)]. It is a 191-item parent-report form of temperament designed for use with children between ages 3 and 12 months. The IBQ-R yields 14 scales that form three higher-order factors (Montirosso et al., 2011): a Positive Affectivity/Surgency factor, containing approach, vocal reactivity, high-intensity pleasure, smiling and laughter, activity level, and perceptual sensitivity subscales; a Negative Affectivity factor, including sadness, distress to limitations, fear, and low falling reactivity subscales; and an Orienting/Regulatory Capacity factor, including low-intensity pleasure, cuddliness, duration of orienting, and soothability subscales.

Mothers and fathers rated the frequency of infant behaviors on a scale from 1 (never) to 7 (always). In the current study, the internal consistency coefficient was calculated for the three overarching factor scores of the IBQ-R for both mothers and fathers.

The alpha coefficients for Positive Affectivity/Surgency were α = 0.74 (Time 1) and α = 0.73 (Time 2) for the mothers; for the fathers, they were α = 0.76 (Time 1) and α = 0.73 (Time 2).

The alpha coefficients for Negative Affectivity were α = 0.72 (Time 1) and α = 0.74 (Time 2) for mothers; for the fathers, they were α = 0.71 (Time 1) and α = 0.73 (Time 2).

The alpha coefficients for Orienting/Regulatory Capacity were α = 0.72 (Time 1) and α = 0.75 (Time 2) for the mothers; for the fathers, they were α = 0.71 (Time 1) and α = 0.74 (Time 2).

The IBQ-R factor scale scores showed stability from 3 to 12 months (*r* = 0.55, *p* < 0.001, for Positive Affectivity/Surgency; *r* = 54, *p* < 0.001, for Negative Affectivity; and *r* = 0.61, *p* < 0.001 for Orienting/Regulatory Capacity). In addition, the Positive Affectivity/Surgency and Orienting/Regulatory Capacity factor scales are intercorrelated with each other (*r* = 0.48, *p* < 0.001, at Time 1 and *r* = 0.58, *p* < 0.001, at Time 2).

### Procedure

The study design attained approval from academy ethics committees. All parents signed a written informed consent questionnaire and received an informative sheet on the investigation.

At 3 months after their child’s birth (Time 1), mothers and fathers independently filled out: the STAI (Spielberger et al., 1983), EPDS (Cox et al., 1987), and IBQ-R (Gartstein and Rothbart, 2003). At 12 months after their child’s birth (Time 2), mothers and fathers were again asked to fill out the EPDS, the STAI, and the IBQ-R.

### Data Analysis

The data were preliminarily examined for errors and outliers. Preliminary analysis showed that data were mostly complete for both mother and father variables. Specifically, there were no missing data for mothers’ and fathers’ age, mothers’ and fathers’ educational levels, and mothers’ EPDS and STAI scores at Time 1. The following measures had missing data for fewer than 3% of the participants: fathers’ EPDS, STAI, and IBQR scores at Time 1, mothers’ EPDS and STAI at Time 2, and IBQR scores at Time 1 and Time 2. Finally, fathers’ EPDS, STAI, and IBQR scores at Time 2 had 4–5% of missing data. Missing data were corrected with mean imputation in total scores of scales (Graham, 2009).

Differences between mothers and fathers in anxiety, depression, and perception of infant temperament were investigated through a series of Student’s paired *t*-tests.

Repeated-measures analysis of variance (ANOVA) was applied to evaluate differences in temperament between boys and girls at each time of assessment. Specifically, for each IBQ-R factor scale and each temperament scale score, a within–between repeated-measures ANOVA was run to examine between-group (boys *vs.* girls) differences in temperament by time of evaluation (Time 1 and Time 2). To evaluate significant effects, Bonferroni correction for multiple comparisons was applied.

Effect sizes were estimated using Cohen’s *d* for *t*-test and partial eta squared (η*_*p*_*^2^) for ANOVA (Gravetter and Wallnau, 2006).

Pearson correlation coefficients were calculated to test bivariate associations between the mothers’ or fathers’ anxiety/depressive symptoms and the perceived infants’ temperament scores at both Times 1 and 2.

## Results

### Differences Between Fathers’ and Mothers’ Postnatal Anxiety and Depression Symptoms and Perception of Infant Temperament

Table 1 presents the statistical results of the paired comparison *t*-test.

**TABLE 1:** Descriptive statistics and parents’ comparisons for the EPDS, STAI, and IBQ-R factor and dimension scores.

	Time 1					Time 2				
							
	Mothers	Fathers				Mothers	Fathers			
	*N* = 94	*N* = 94				*N* = 94	*N* = 94			

	Mean score (SD)	Mean score (SD)	*T*	*p*	*d*	Mean score (SD)	Mean score (SD)	*t*	*p*	*d*
**EPDS**	**6.24 (4.10)**	**4.15 (2.81)**^a^	**4**.**52**	**0.000**	**0.46**	**5.93 (3.32)**^a^	**3.32 (2.73)**^b^	**6.96**	**0.000**	**0.72**
**STAI—State**	**33.60 (7.16)**	**31.67 (5.96)**^a^	**2**.**32**	**0.02**	**0.24**	**34.99 (8.36)**^a^	**30.76 (6.22)**^d^	**4.61**	**0.000**	**0.48**
**STAI—Trait**	**33.55 (6.75)**	**31.63 (6.54)**^a^	**2**.**19**	**0.03**	**0.23**	**33.39 (6.68)**^a^	**31.06 (5.75)**^d^	**2.96**	**0.004**	**0.31**
**IBQ-R—PAS**	**4.24 (0.69)**^b^	**4.16 (0.75)**^a^	**1**.**11**	**0.27**	**0.08**	**4.97 (0.53)**^c^	**4.86 (0.52)**^e^	**1.72**	**0.09**	**0.18**
Approach	4.45 (1.20)^b^	4.27 (1.17) ^*c*^	1.43	0.16	0.15	5.44 (0.85)^c^	5.21 (0.94)^e^	2.29	0.03	0.23
Vocal reactivity	4.36 (1.00)^b^	4.20 (1.00)^c^	1.33	0.19	0.14	5.13 (0.81)^c^	4.92 (0.78)^e^	2.23	0.03	0.23
High pleasure	5.25 (1.10)^b^	5.11 (1.11)^c^	1.13	0.26	0.12	6.09 (0.6)^c^	5.80 (0.79)^e^	2.63	0.002	0.33
Smiling and laughter	4.27 (1.00)^b^	4.36 (1.01)^c^	−0.84	0.41	0.09	4.67 (0.86)^c^	4.61 (0.9)^e^	0.61	0.54	0.07
Activity level	3.61 (0.81)^b^	3.41 (0.80)^c^	2.25	0.03	0.24	4.01 (0.81)^c^	3.98 (0.89)^e^	0.30	0.77	0.03
Perceptual sensitivity	3.52 (0.86)^b^	3.58 (0.77)^c^	−0.37	0.69	0.06	4.46 (1.05)^c^	4.43 (1.03)^e^	0.20	0.85	0.02
**IBQ-R—NA**	**3.22 (0.48)**^a^	**3.28 (0.49)**^b^	**−0**.**90**	**0.37**	**0.11**	**3.58 (0.53)**^c^	**3.54 (0.51)**^e^	**0.83**	**0.41**	**0.08**
Sadness	2.95 (0.99)^a^	2.89 (0.82)^b^	0.53	0.60	0.06	2.97 (0.78)^c^	2.95 (0.9)^e^	0.26	0.80	0.02
Distress to limitations	3.31 (0.79)^a^	3.24 (0.77)^b^	0.98	0.33	0.10	3.97 (0.86)^c^	3.78 (0.78)^e^	2.32	0.02	0.24
Fear	2.13 (0.72)^a^	2.25 (0.77)^b^	−1.32	0.19	0.14	2.76 (0.83)^c^	2.87 (0.87)^e^	−1.23	0.22	0.12
Falling reactivity	4.51 (1.07)^a^	4.51 (1.17)^b^	−0.03	0.98	0.00	4.47 (1.10)^c^	4.56 (1.02)^e^	−0.81	0.42	0.08
**IBQ-R—ORC**	**4.78 (0.63)**^a^	**4.67 (0.68)**^b^	**1**.**55**	**0.12**	**0.16**	**4.73 (0.61)**^c^	**4.73 (0.63)**^e^	−**0.06**	**0.95**	**0.00**
Low pleasure	5.30 (1.00)^a^	5.10 (0.99)^b^	1.84	0.07	0.18	5.02 (1.00)^c^	5.10 (1.17)^e^	−0.68	0.50	0.07
Cuddliness	5.28 (1.16)^a^	5.22 (1.13)^b^	0.92	0.36	0.06	5.23 (1.03)^c^	5.11 (1.06)^e^	1.39	0.17	0.14
Duration of orienting	4.01 (1.00)^a^	3.93 (1.16)^b^	0.57	0.57	0.06	3.85 (0.96)^c^	4.00 (0.99)^e^	−1.30	0.20	0.14
Soothability	4.54 (0.85)^a^	4.49 (1.03)^b^	0.45	0.66	0.05	4.80 (0.95) ^*c*^	4.71 (0.91)^e^	0.99	0.32	0.10

*PAS = Positive Affectivity/Surgency; NA = Negative Affectivity; ORC = Orienting/Regulatory Capacity; EPDS = Edinburgh Postnatal Depression Scale; STAI = State–Trait Anxiety Inventory (STAI); IBQ-R = Infant Behavior Questionnaire. Results for EPDS, STAI scores, and IBQR factor scores shown in bold. ^*a*^ One participant had missing value(s) on only one or two items. ^*b*^ Two participants had missing value(s) on only one or two items. ^*c*^ Three participants had missing value(s) on only one or two items. ^*d*^ Four participants had missing value(s) on only one or two items. ^*e*^ Five participants had missing value(s) on only one or two items.*

The results showed that mothers showed more depressive symptoms than their partners at both times (Time 1 and Time 2). However, the mean scores for both mothers and fathers were under the cutoff point for risk of clinical depression. Similarly, mothers showed more anxious symptoms than fathers at both times. Nevertheless, in this case, the mean scores for both mothers and fathers were under the STAI cutoff.

As concerns IBQ-R, differences between the mothers and fathers within each couple were found with respect to the specific dimensions of the infants’ Positive Affectivity/Surgency and Negative Affectivity. Specifically, at Time 1, the mothers perceived their infant as having more motor activity compared to their partners. Also, at Time 2, the mothers perceived their infant as more positively responsive in terms of their levels of high-intensity pleasure, vocal reactivity, approach, excitement, and positive anticipation of pleasurable activities compared to fathers.

### The Developmental Change and Gender Differences in Perceived Infant Temperament

A 2 (gender: boys *vs.* girls) × 2 (time: Time 1 *vs.* Time 2) repeated-measures ANOVA was performed for each IBQR factor scale and subscale score.

Table 2 presents the statistical results of all main effects for gender, age, and interactions for each factor scale and IBQR subscale score. Means and SDs for all factor and subscales scores at Time 1 and Time 2 for boys and girls are shown in Table 3.

**TABLE 2:** Effects of gender, children’s age, and their interactions on IBQ-R factors and dimensions.

							Interactions of		
							
	Gender			Children’s age			Gender × Children’s age		
			
Mothers	*F*	*p*	η *_*p*_*^2^	*F*	*p*	η *_*p*_*^2^	*F*	*p*	η *_*p*_*^2^

PAS	0.01	0.94	0.00	131.03	0.000	0.59	1.75	0.19	0.19
**Approach**	0.04	0.85	0.00	62.72	0.000	0.41	0.08	0.78	0.00
**Vocal reactivity**	0.31	0.58	0.00	60.88	0.000	0.40	0.13	0.72	0.00
**High pleasure**	2.68	0.11	0.03	49.49	0.000	0.35	0.43	0.51	0.01
**Smiling and laughter**	0.02	0.90	0.00	12.09	0.001	0.12	1.43	0.23	0.02
**Activity level**	0.57	0.45	0.01	12.75	0.001	0.12	7.37	0.01	0.07
**Perceptual sensitivity**	0.94	0.34	0.01	63.33	0.000	0.41	1.94	0.17	0.02
**NA**	**4.45**	**0.04**	**0.05**	**48.61**	0.000	**0.35**	**0.00**	**0.95**	**0.00**
**Sadness**	0.94	0.34	0.01	0.60	0.81	0.00	0.43	0.51	0.01
**Distress to limitations**	0.90	0.35	0.01	63.53	0.000	0.41	0.86	0.36	0.01
**Fear**	13.00	0.001	0.12	50.45	0.000	0.35	0.01	0.94	0.00
**Falling reactivity**	0.17	0.69	0.00	0.20	0.66	0.00	0.46	0.50	0.01
**ORC**	**2.53**	**0.12**	**0.03**	**1.33**	**0.25**	**0.01**	**0.43**	**0.52**	**0.01**
**Low pleasure**	0.05	0.83	0.00	7.01	0.01	0.07	1.47	0.23	0.02
**Cuddliness**	9.16	0.003	0.09	0.12	0.74	0.00	0.19	0.66	0.00
**Duration of orienting**	0.02	0.89	0.00	2.74	0.10	0.03	0.05	0.82	0.00
**Soothability**	1.10	0.30	0.01	9.30	0.003	0.09	1.03	0.31	0.01

**Fathers**	***F***	***p***	**η *_*p*_*^2^**	***F***	***p***	**η *_*p*_*^2^**	***F***	***p***	**η *_*p*_*^2^**

**PAS**	**0.16**	**0.69**	**0.00**	**88.50**	**0.000**	**0.5**	**0.31**	**0.58**	**0.00**
Approach	0.01	0.93	0.00	61.30	0.000	0.4	0.88	0.35	0.01
Vocal reactivity	1.47	0.23	0.02	46.02	0.000	0.3	0.19	0.67	0.00
High pleasure	8.02	0.006	0.10	29.58	0.000	0.2	0.16	0.69	0.00
Smiling and laughter	0.04	0.84	0.00	6.97	0.01	0.1	0.10	0.75	0.00
Activity level	1.73	0.47	0.02	22.94	0.001	0.2	3.76	0.67	0.04
Perceptual sensitivity	0.04	0.84	0.00	48.60	0.000	0.3	0.32	0.57	0.00
**NA**	**0.83**	**0.36**	**0.01**	**19.82**	**0.000**	**0.2**	**0.66**	**0.42**	**0.01**
Sadness	0.00	0.97	0.00	0.25	0.62	0.000	0.00	1.00	0.00
Distress to limitations	0.59	0.45	0.01	33.80	0.000	0.30	0.15	0.70	0.00
Fear	2.83	0.10	0.03	27.41	0.000	0.2	1.1	0.30	0.01
Falling reactivity	1.00	0.32	0.01	0.21	0.65	0.02	0.61	0.44	0.01
**ORC**	**1.90**	**0.17**	**0.02**	**0.56**	**0.46**	**0.01**	**0.04**	**0.84**	**0.00**
Low pleasure	0.70	0.41	0.01	0.01	0.94	0.00	0.41	0.52	0.00
Cuddliness	8.10	0.005	0.10	0.41	0.53	0.00	0.13	0.72	0.00
Duration of orienting	0.36	0.55	0.00	0.26	0.61	0.00	0.08	0.78	0.00
Soothability	0.54	0.43	0.01	3.62	0.06	0.04	0.05	0.83	0.00

*2 (gender) × 2 (time) repeated-measures ANOVA results. Results for IBQR factor scores shown in bold. Error degrees of freedom for *F* statistics were 1.92 for the three factor scales and for the 14 subscales.*

**TABLE 3:** Means and SDs of IBQ-R factor and dimension scores by children’s age (Time 1 and Time 2) and gender.

	Children’s age		Gender			
		
	Time 1	Time 2	Time 1		Time 2	

Mothers	Total sample mean score *(SD)*	Total sample mean score *(SD)*	Boys *(N = 52)* Mean score *(SD)*	Girls *(N = 42)* Mean score *(SD)*	Boys *(N = 52)* Mean score *(SD)*	Girls *(N = 42)* Mean score *(SD)*
**PAS**	**4,24 (0.69)**	**4.97 (0.53)**	**4.2 (0.6)**	**4.3 (0.8)**	**5.0 (0.5)**	**4.9 (0.5)**
Approach	4.45 (1.20)	5.44 (0.85)	4.5 (1.1)	4.4 (1.3)	5.4 (0.9)	5.4 (0.8)
Vocal reactivity	4.36 (1.00)	5.13 (0.81)	4.3 (1.1)	4.4 (0.9)	5.1 (0.9)	5.2 (0.7)
High pleasure	5.25 (1.10)	6.09 (0.6)	5.4 (0.9)	5.1 (1.3)	6.1 (0.5)	6.0 (0.7)
Smiling and laughter	4.27 (1.00)	4.67 (0.86)	4.2 (1.0)	4.4 (1.0)	4.7 (0.9)	4.6 (0.8)
Activity level	3.61 (0.81)	4.01 (0.81)	3.5 (0.7)	3.7 (0.9)	4.2 (0.9)	3.8 (0.6)
Perceptual sensitivity	3.52 (0.86)	4.46 (1.05)	3.4 (1.2)	3.7 (1.2)	4.4 (1.0)	4.5 (1.1)
**NA**	3.22 (0.48)	3.58 (0.53)	**3.1 (0.5)**	**3.3 (0.5)**	**3.5 (0.6)**	**3.7 (0.5)**
Sadness	2.95 (0.99)	2.97 (0.78)	2.9 (0.9)	3.0 (1.1)	2.9 (0.8)	3.1 (0.7)
Distress to limitations	3.31 (0.79)	3.97 (0.86)	3.3 (0.8)	3.3 (0.8)	3.9 (0.9)	4.1 (0.7)
Fear	2.13 (0.72)	2.76 (0.83)	1.9 (0.5)	2.4 (0.8)	2.5 (0.8)	3.0 (0.8)
Falling reactivity	4.51 (1.07)	4.47 (1.10)	4.4 (1.1)	4.6 (1.1)	4.5 (1.2)	4.5 (1.0)
**ORC**	**4.78 (0.63)**	**4.73 (0.61)**	**4.7 (0.6)**	**4.9 (0.7)**	**4.7 (0.6)**	**4.8 (0.6)**
Low pleasure	5.30 (1.00)	5.02 (1.00)	5.3 (1.0)	5.4 (1.0)	5.1 (1.0)	4.9 (1.0)
Cuddliness	5.28 (1.16)	5.23 (1.03)	5.1 (1.2)	5.6 (1.1)	5.0 (1.0)	5.6 (1.0)
Duration of orienting	4.01 (1.00)	3.85 (0.96)	4.0 (1.0)	4.0 (1.0)	3.8 (0.9)	3.9 (1.1)
Soothability	4.54 (0.85)	4.80 (0.95)	4.4 (0.8)	4.7 (1.0)	4.8 (1.0)	4.8 (0.9)

	***Time 1***	***Time 2***	***Time 1***		***Time 2***	

**Fathers**	**Total sample mean score *(SD)***	**Total sample mean score *(SD)***	**Boys *(N = 52)* Mean score *(SD)***	**Girls *(N = 42)* Mean score *(SD)***	**Boys *(N = 52)* Mean score *(SD)***	**Girls *(N = 42)* Mean score *(SD)***

**PAS**	**4.16 (0.75)**	**4.86 (0.52)**	**4.2 (0.8)**	**4.1 (0.7)**	**4.9 (0.5)**	**4.9 (0.5)**
Approach	4.27 (1.17)	5.21 (0.94)	4.3 (1.1)	4.2 (1.2)	5.2 (0.9)	5.3 (0.9)
Vocal reactivity	4.20 (1.00)	4.92 (0.78)	4.1 (1.1)	4.3 (0.9)	4.8 (0.8)	5.0 (0.8)
High pleasure	5.11 (1.11)	5.80 (0.79)	5.3 (1.0)	4.9 (1.2)	6.0 (0.7)	5.6 (0.8)
Smiling and laughter	4.36 (1.01)	4.61 (0.9)	4.4 (1.0)	4.3 (1.0)	4.6 (0.9)	4.6 (0.9)
Activity level	3.41 (0.80)	3.98 (0.89)	3.4 (0.8)	3.4 (0.8)	4.1 (0.9)	3.8 (0.8)
Perceptual sensitivity	3.58 (0.77)	4.43 (1.03)	3.6 (1.2)	3.6 (1.1)	4.4 (1.1)	4.5 (0.9)
**NA**	3.28 (0.49)	3.54 (0.51)	**3.2 (0.5)**	**3.3 (0.5)**	**3.5 (0.6)**	**3.6 (0.4)**
Sadness	2.89 (0.82)	2.95 (0.9)	2.9 (0.8)	2.9 (0.8)	2.9 (1.0)	2.9 (0.8)
Distress to limitations	3.24 (0.77)	3.78 (0.78)	3.2 (0.8)	3.3 (0.8)	3.7 (0.9)	3.8 (0.7)
Fear	2.25 (0.77)	2.87 (0.87)	2.1 (0.6)	2.4 (0.9)	2.8 (1.0)	2.9 (0.7)
Falling reactivity	4.51 (1.17)	4.56 (1.02)	4.6 (1.1)	4.4 (1.2)	4.6 (1.1)	4.5 (0.9)
**ORC**	**4.67 (0.68)**	**4.73 (0.63)**	**4.6 (0.7)**	**4.8 (0.7)**	**4.7 (0.7)**	**4.8 (0.6)**
Low pleasure	5.10 (0.99)	5.10 (1.17)	5.1 (1.1)	5.1 (0.9)	5.2 (1.3)	5.0 (1.0)
Cuddliness	5.22 (1.13)	5.11 (1.06)	4.9 (1.2)	5.5 (1.0)	4.8 (1.1)	5.4 (1.0)
Duration of orienting	3.93 (1.16)	4.00 (0.99)	3.9 (1.1)	4.0 (1.2)	4.0 (0.9)	4.0 (1.1)
Soothability	4.49 (1.03)	4.71 (0.91)	4.4 (1.0)	4.5 (1.0)	4.6 (0.9)	4.8 (0.9)

*Results for IBQR factor scores shown in bold.*

#### Mothers

Gender effects showed higher fear and cuddliness scores in girls: mothers perceived girls as more distressed in response to unexpected changes in stimulation as well as more positively responsive in terms of their levels of cuddliness compared to boys.

Age differences indicated that between 3 and 12 months of age, mothers perceive that their infants become higher on Positive Affectivity/Surgency and on all the subscales contributing to this factor as well as on Negative Affectivity and the associated subscales of distress to limitation and fear. No age effects were found for Orienting/Regulatory Capacity except for low pleasure and soothability subscales; mothers perceive that their infants become lower in low pleasure and higher on soothability.

Gender × children’s age interactions were significant for the activity level subscale. This significant interaction was interpreted using a follow-up test that examined the effect separately at Time 1 and Time 2. Results showed that at 12 months of age, mothers perceive boys as being characterized by more locomotor activity, movement of the arms and legs, and squirming than girls, *t* (92) = 2.33, *p* = 0.02, *d* = 0.49.

#### Fathers

As concerns fathers, gender effects showed higher high pleasure scores in boys and higher cuddliness scores in girls.

Fathers perceive their boys as more positively responsive in terms of their levels of high-intensity pleasure compared to girls, while they perceive girls as more positively responsive in terms of their level of cuddliness compared to boys.

Age differences indicated that between Time 1 and Time 2, fathers perceive that their infants become higher on Positive Affectivity/Surgency and on all subscales contributing to this factor as well as on Negative Affectivity and on the associated subscales of distress to limitation and fear. No age effects were found for Orienting/Regulatory Capacity and the associated subscales.

Finally, we did not find significant interactions between Gender and Children’s Age for any factor or subscale.

### Associations Between Parent’s Postnatal Anxiety and Depression and Perceived Infant’s Temperament

Since the IBQ-R involves so many subscales, we examined the relationship between the EPDS and STAI scores and the IBQ-R factor scores. Pearson correlations between maternal and paternal measures and IBQ-R factors scores at Times 1 and 2 are shown in Table 4.

**TABLE 4:** Pearson correlations between maternal and paternal measures and IBQ-R factor scales.

IBQ-R factor scales						

Maternal measures	PAS Time 1	NA Time 1	ORC Time 1	PAS Time 2	NA Time 2	ORC Time 2

Maternal measures	PAS Time 1	NA Time 1	ORC Time 1	PAS Time 2	NA Time 2	ORC Time 2
EPDS Time 1	-.208*	.285**	-.131	-.245*	.225*	-.172
STAI—STATE Time 1	-.075	.280**	-.119	-.259*	.206*	-.292**
STAI—TRAIT Time 1	-.066	.262*	-.076	-.255*	.240*	-.225*
EPDS Time 2	-.100	.223*	-.084	-.222*	.267**	-.150
STAI—STATE Time 2	.016	.151	-.039	-.197	.250*	-.212*
STAI—TRAIT Time 2	-.099	.196	-.117	-.243*	.236*	-.255*

IBQ-R factor scales						

***Paternal measures***	**PAS Time 1**	**NA Time 1**	**ORC Time 1**	**PAS Time 2**	**NA Time 2**	**ORC Time 2**

EPDS Time 1	-.085	.289**	-.111	-.051	.219*	-.115
STAI—STATE Time 1	.063	.296**	-.041	-.049	.272**	-.060
STAI—TRAIT Time 1	-.010	.291**	.002	.026	.204*	-.016
EPDS Time 2	-.048	.255*	-.144	-.020	.209*	-.102
STAI—STATE Time 2	.049	.161	.004	.024	.252*	-.023
STAI—TRAIT Time 2	-.148	.337**	-.108	-.126	.291**	-.170

*p < 0.05; **p < 0.01.

#### Mothers

As concerns mothers, the results showed that maternal anxiety and depression symptoms had a negative significant relationship with Positive Affectivity/Surgency and a positive significant relationship with Negative Affectivity at both times, whereas no significant correlations were observed between maternal depression and the Orienting/Regulatory Capacity factor scale at both times; instead, maternal anxiety had a significant relationship with Orienting/Regulatory Capacity at Time 2.

#### Fathers

As concerns fathers, the results showed that paternal anxiety and depression symptoms had a positive significant relationship with Negative Affectivity at both times, whereas no significant correlations were observed between paternal anxiety and depression and the Positive Affectivity/Surgency and Orienting/Regulatory Capacity scores at both times (Table 4).

## Discussion

The main objective of our study was to assess differences and similarities between mothers’ and fathers’ perception of their infant’s temperament, to assess if the perception of infant temperament is different in reference to the age and gender of the baby and to evaluate the association between parents’ postnatal anxiety and depression symptoms and the perception of their infants’ temperament.

### Differences and Similarities Between Mothers’ and Fathers’ Postnatal Anxiety and Depression Symptoms and the Perception of Their Infant’s Temperament at Time 1 and Time 2

We have confirmed that women presented higher scores of self-reported postnatal anxiety and depression compared to men, indicating that mothers, more than fathers, are at higher risk to develop affective issues after the birth of their child (Matthey et al., 2003; Cameron et al., 2016; Vismara et al., 2016).

In general, mothers and fathers would give similar descriptions of their child’s temperament throughout the first year of life, although a few noteworthy differences emerged. Specifically, at 3 months of age, the mothers perceive their infant as having more motor activity compared to their partners. This result may be explained by the fact that mothers are the primary caregiver in the earliest stages of life of their babies. Mothers are the person who is mainly engaged in her baby’s physical care and rearing and need to respond to their infants’ signals that they are learning to interpret. With time, mothers are more capable of acknowledging their child’s demands, and the interactions are easier and more meaningful. Indeed, at 12 months of age, the mothers perceive their infant as more positively responsive in terms of their levels of high-intensity pleasure, vocal reactivity, approach, excitement, and positive anticipation of pleasurable activities compared to fathers, who are at the beginning of their active involvement with their infant, who is, for her/his part, developing more and more social and interactive skills. These results are in line with other research (Prino et al., 2016; Vismara et al., 2016; Rollè et al., 2017).

### Developmental Change and Gender Differences in Perceived Infant Temperament

In our group, most of the perceived temperamental characteristics showed developmental change for both mothers and fathers, as reported by other scholars (Planalp et al., 2013; Laceulle et al., 2014; Zhang et al., 2018). Also, in our sample, the global factors of Positive Affectivity/Surgency and Negative Affectivity increased. We also highlight the increase in the following subscales: activity level, distress to limitation, fear, smiling and laughter, high pleasure, perceptual sensitivity, approach, and vocal reactivity. Instead, only mothers reported a higher level of soothability and a decrease of low pleasure. The remaining subscales did not show significant variations but, rather, stability. These data seem to confirm how in early infancy, motor activity and the expression of positive and negative affectivity are crucial features of this developmental stage (Gartstein and Rothbart, 2003; Rothbart and Gartstein, 2008). Indeed, children’s improvements in motor skills are matched with more sophisticated cognitive abilities that allow better emotional and behavioral control (Planalp et al., 2017). Furthermore, temperament itself, although biologically based, may be changed by the interaction of the children with their milieu (Rothbart, 2011; Perry et al., 2018); therefore, environmental epigenetics processes may account for temperamental characteristics and their lack of stability (Gartstein and Skinner, 2018).

Additionally, we found, also in accordance with other research (Ready et al., 2005; Else-Quest et al., 2006; Cameron Ponitz et al., 2008; Else-Quest, 2012; Coe et al., 2020), differences in relation to the gender of parents and specific temperamental characteristics of girls and boys.

In particular, both mothers and fathers perceive girls as more positively responsive in terms of their levels of cuddliness compared to boys.

The mothers perceive girls as more distressed in response to unexpected changes in stimulation, novel physical objects, or social stimuli than boys. Also, only at 12 months of age, the mothers perceive boys as characterized by greater motor activity, squirming, and locomotor movement.

Fathers instead perceive boys as more positively responsive in terms of their levels of enjoyment associated with high stimulus intensity, rate, complexity, novelty, and incongruity compared to girls.

These data corroborate several scholars who proved that parents tend to behave differently with sons and daughters. These behaviors and attitudes appear to be neurobiologically grounded (Mascaro et al., 2017), as well as culturally defined (Lipowska et al., 2016). However, it is important to underline that both child and parent gender as well as dispositional characteristics may mutually influence the parent–child relationship (Sameroff, 2010).

### Association Between Parents’ Postnatal Anxiety and Depression Symptoms and the Perception of Their Infants’ Temperament

Maternal and paternal anxiety and depression symptoms are associated with infants’ negative affectivity defined in terms of fear, sadness, frustration, and discomfort. Mothers with high anxiety and depression levels perceived their infants as having a lower tendency to approach novelty, to search for environmental stimulation, and to display and feel positive emotions. Finally, only at 12 months of age of the infants, maternal anxiety is negatively associated with infant duration of orienting attention, soothability, cuddliness, and enjoyment of low-intensity activities (Hanington et al., 2010; Prino et al., 2016).

These findings confirm that mothers who report prenatal and postnatal anxiety and depression tend to perceive their infants as fussier, slower to adapt to novelty, and more difficult than control groups (Austin et al., 2005; Blair et al., 2011). Less is known on fathers; however, the existing data show a similar association as in mothers: higher levels of reported depressive and anxiety symptoms are related to the perception of a more difficult-tempered infant, who is described as substantially fussier (Davé et al., 2005; Kerstis et al., 2013). We believe that these difficulties arise from the quality of the caregiving relationship in the context of parental anxiety and depressive symptoms. Interestingly, Hanington et al. (2010) found that fathers’ symptoms were significantly associated only with male children’s temperament.

Indeed, it is now consistently proven that boys exhibit higher activity levels and need their caregiver to function as an external regulator, while girls show higher shyness but better self-soothing abilities (Planalp et al., 2017; Arace et al., 2019). Certainly, we may then conceive emotion regulation as a central aspect of temperament, which is, in turn, linked to the risk of developing psychological difficulties (Gartstein and Skinner, 2018). Typically, girls show more internalizing problems, whereas boys have higher externalizing problems (Letourneau et al., 2019). However, such outcomes must consider the transactional processes among genes and environment, especially parental characteristics and caregiving abilities.

### Limitations

Some limitations of the present study should be observed. First, maternal and paternal anxiety and depressive symptoms were self-reported; therefore, findings must be interpreted with caution in terms of the association between parental mental health problems and child temperament.

Indeed, the participants were all primiparous parents belonging to a non-clinical, low-psychosocial-risk sample; therefore, the generalizability of our results needs further confirmation.

Furthermore, child temperament is assessed in terms of parental perception, and it could be influenced by their mental health. However, it is important to consider these subjective experiences. Indeed, an active and challenging baby may be particularly difficult for a parent who is depressed and/or anxious, increasing the likelihood of poor outcomes for both the child and the parents (Austin et al., 2005). Also, even if there is some overlapping between parental perception and a professional’s observation of infant behavior (Planalp et al., 2017), both methods should be included in future studies to give a more reliable picture of the child’s characteristics.

Besides, gender differences in temperament may be influenced by moderating factors. Variables such as cultural and socioeconomic contexts, or participants belonging to a special population should be included in future studies. In a longitudinal perspective, the relation between parental characteristics – (such as parental stress, coping strategies, dyadic adjustment, and parental practices –), clinical depression/anxiety, and child temperament should be deepened.

Finally, future studies would benefit of from evaluating, within the above- described multifaceted perspective, the complex interplay between biology and environment and the possible existing relation between some specific genes, perinatal aspects, and infant temperament, as underlined by Gu et al. (2019).

## Conclusion

An adequate, mutual interaction between environment and temperament favors the best developmental outcomes in children (Thomas and Chess, 1975). However, parents may adjust to their children’s characteristics. When a parents is are not able to adjust to their infant’s needs due to mental health and personal and contextual strains, their offspring may be at risk for developmental perturbations like behavioral and cognitive difficulties (Rollè et al., 2019).

Following the literature, we know that mother’s and father’s depression can influence their parenting, the relationship with their infant, and the latter’s temperament (Hanington et al., 2010).

Thus, providing support not only for mothers but also for fathers in the postnatal period may be an effective prevention strategy to enhance child’s development.

Regarding intervention strategies, it could be important to prevent dysfunctional parents—infant relationships by means of home-visiting programs (van Doesum et al., 2008) that aim to reduce risk factors for the child’s mental health as well as to enhance protective factors and resilience (Gelfand et al., 1996; Cicchetti et al., 2000). In particular, home-visiting mentalization-based interventions may contribute to improving parental depressive and anxiety symptoms, affective responsivity, and parent/child communicative exchanges, aiding in the prevention of negative developmental outcomes (Fonagy et al., 2002; Slade et al., 2005; Vismara et al., 2020).

## Data Availability Statement

The datasets presented in this article are not readily available because our data are identifiable. Requests to access the datasets should be directed to cristina.sechi@unica.it.

## Ethics Statement

The studies involving human participants were reviewed and approved by the Comitato Etico del Dipartimento di Pedagogia, Psicologia, Filosofia dell’Università degli Studi di Cagliari. Written informed consent to participate in this study was provided by the participants’ legal guardian/next of kin. Written informed consent was obtained from the individual(s), and minor(s)’ legal guardian/next of kin, for the publication of any potentially identifiable images or data included in this article.

## Author Contributions

CS contributed to organize the recruitment of the sample, prepared data set, performed statistical analyses, and contributed to all the sections of the manuscript. LV contributed to prepare the study design, organize the recruitment of the sample, and writing all the sections of the manuscript. LR and LP contributed to organize the recruitment of the sample and writing the manuscript s Introduction, Discussion, and References sections. LL contributed to prepare the study design, organize the recruitment of the sample, and supervised data collection and the research team. All authors reviewed and approved manuscript for publication.

## Conflict of Interest

The authors declare that the research was conducted in the absence of any commercial or financial relationships that could be construed as a potential conflict of interest.
